# Human motor cortical gamma activity relates to GABAergic intracortical inhibition and motor learning

**DOI:** 10.1162/imag_a_00538

**Published:** 2025-04-24

**Authors:** Catharina Zich, Magdalena Nowak, Emily L. Hinson, Camille Lasbareilles, Valentina Mancini, Alek Pogosyan, Oana Puicar, Ioana-Florentina Grigoras, Patricia Cambalova, Jacqueline Scholl, Laurie Josephs, Andrew J. Quinn, Mark W. Woolrich, Charlotte J. Stagg

**Affiliations:** Oxford Centre for Human Brain Activity, Wellcome Centre for Integrative Neuroimaging, Department of Psychiatry, University of Oxford, Oxford, United Kingdom; Wellcome Centre for Integrative Neuroimaging, FMRIB, Nuffield Department of Clinical Neurosciences, University of Oxford, Oxford, United Kingdom; MRC Brain Network Dynamics Unit, Nuffield Department of Clinical Neurosciences, University of Oxford, Oxford, United Kingdom; Division of Neuroscience and Experimental Psychology, University of Manchester, Manchester, United Kingdom; Lyon Neuroscience Research Center, INSERM U1028, CNRS UMR5292, PSYR2 Team, University Lyon, Lyon, France; Centre Hospitalier Le Vinatier, Pôle EST, Bron, France; Centre for Human Brain Health, School of Psychology, University of Birmingham, Birmingham, United Kingdom

**Keywords:** GABA, MEG, motor learning, motor system, TMS

## Abstract

Gamma activity (γ, >30 Hz) is universally demonstrated across brain regions and species. However, the physiological basis and functional role of γ sub-bands (slow-γ, mid-γ, fast-γ) have been predominantly studied in rodent hippocampus; γ activity in the human neocortex is much less well understood. We use electrophysiology, non-invasive brain stimulation, and several motor tasks to examine the properties of sensorimotor γ activity sub-bands and their relationship with both local GABAergic activity and motor learning. Data from three experimental studies are presented. Experiment 1 (N = 33) comprises magnetoencephalography (MEG), transcranial magnetic stimulation (TMS), and a motor learning paradigm; experiment 2 (N = 19) uses MEG and motor learning; and experiment 3 (N = 18) uses EEG and TMS. We characterised two distinct γ sub-bands (slow-γ, mid-γ) which show a movement-related increase in activity during unilateral index finger movements and are characterised by distinct temporal–spectral–spatial profiles. Bayesian correlation analysis revealed strong evidence for a positive relationship between slow-γ (~30–60 Hz) peak frequency and GABAergic intracortical inhibition (as assessed using the TMS-metric short interval intracortical inhibition). There was also moderate evidence for a relationship between the power of the movement-related mid-γ activity (60–90 Hz) and motor learning. These relationships were neurochemical and frequency specific. These data provide new insights into the neurophysiological basis and functional roles of γ activity in human M1 and allow the development of a new theoretical framework for γ activity in the human neocortex.

## 1 Introduction

Activity within the gamma band (γ, >30 Hz) has been observed in several cortical and subcortical structures in the mammalian brain (Buzsáki & Wang, 2012). To date, γ activity has been best explored in the rodent hippocampus, where this broad frequency range has been subdivided into slow-γ (~30–50 Hz), mid-γ (~50–100 Hz), and fast-γ (~100–140 Hz) activity (Belluscio et al., 2012;Colgin et al., 2009;Csicsvari et al., 2003;Lasztóczi & Klausberger, 2016). It has been shown that the three γ sub-bands arise from separate locations (Lasztóczi & Klausberger, 2016;Schomburg et al., 2014), reflect distinct microcircuits (Bragin et al., 1995;Colgin et al., 2009;Fernández-Ruiz et al., 2017;Lopes-dos-Santos et al., 2018), and have different functional roles (Carr & Frank, 2012;Colgin, 2015). Considerably less is known about the neurophysiological bases and functional roles of the γ sub-bands in humans and the neocortex.

In the human motor system, a movement-related increase in γ power (γ event-related synchronisation [γ ERS]) has been described (Canolty et al., 2006;Cheyne, 2013;Cheyne et al., 2008;Crone et al., 1998;Muthukumaraswamy, 2010,^2011^;Pfurtscheller & Lopes da Silva, 1999). This has been most frequently reported for the mid-γ band (i.e., mid-γ ERS), shows spatial specificity to the primary motor cortex (M1,Crone et al., 2006) and temporal specificity to the time of the movement. Mid-γ has been suggested to play a role in afferent proprioceptive feedback or relate to more active motor control processes (Miller et al., 2010;Muthukumaraswamy, 2010), and its pro-kinetic role has been demonstrated in several studies (Joundi et al., 2012;Swann et al., 2016,^2018^). In humans, slow-γ has been considerably less well characterised. We attribute this to two factors: first, higher frequencies typically exhibit lower power, making activity at these frequencies less apparent; and second, data are commonly band-pass filtered into the lower frequency range (<30 Hz) and mid-γ range (~60–90 Hz). The few studies that reported movement-related slow-γ activity in the M1 (Crone et al., 1998;Szurhaj et al., 2006) suggested that slow-γ has a distinct spatio-temporal profile and plays a functional role in synchronising the activity of neuronal populations involved in movement.

Slow-γ and mid-γ are likely to share some physiological similarities. Empirical animal studies and computational modelling have demonstrated that GABAergic interneuron-mediated inhibition of pyramidal cell activity generates γ activity in M1 (Buzsáki & Wang, 2012;Gonzalez-Burgos & Lewis, 2008;Sohal et al., 2009). Slow-y frequency has been previously shown to be causally related to local GABAergic synaptic activity in animals (Hájos & Paulsen, 2009;Sohal et al., 2009;Whittington et al., 2000), but this has not been demonstrated in humans. However, in humans, frequency, but not power, of the mid-γ ERS has been found to be related to M1 GABA concentration (Gaetz et al., 2011). Computational work (Brunel & Wang, 2003) and animal studies (Szabadics et al., 2001) further suggest a complex picture between mid-γ peak frequency and GABAergic signalling. Drug studies show that neither diazepam (Hall et al., 2011), tiagabine (Muthukumaraswamy et al., 2013), nor propofol (Saxena et al., 2021) modulate movement-related mid-γ power or frequency in M1. However, diazepam increased power of a broad frequency range including beta, low-γ, and mid-γ in sensorimotor and occipital areas in resting-state data (Hall et al., 2010). Thus, it remains to be determined how closely data from in vitro and invasive in vivo recordings translate into the γ activity seen in human electrophysiological recordings during task and rest.

Given the changes in M1 GABAergic activity during motor learning (Stagg, Bachtiar, et al., 2011),*y*activity may reflect a mechanism by which decreases in local GABAergic signalling mediate behavioural improvements. In line with this hypothesis, our group demonstrated that 75 Hz tACS applied to M1 leads to a significant reduction in GABAergic intracortical inhibition, as assessed by transcranial magnetic stimulation (TMS,Nowak et al., 2017). Moreover, this 75 Hz tACS-induced change in GABAergic intracortical inhibition was closely correlated with an individual’s motor learning ability. Indeed in a very recent study, mid-γ power has been positively related to movement speed, with faster movements associated with greater γ power (Haverland et al., 2024).

In three separate experiments (experiment 1: N = 33; experiment 2: N = 19; experiment 3: N = 18), we characterised sensorimotor γ activity during finger movements using magneto- and electroencephalography (M/EEG) to investigate how an individual’s sensorimotor movement-related γ activity relates to TMS measure of GABAergic intracortical inhibition and the ability to learn a motor task. We hypothesised that properties of the mid-γ motor activity (i.e., power and peak frequency) would correlate positively with our motor learning metric and GABAergic intracortical inhibition (i.e., short interval intracortical inhibition (SICI)). In light of the paucity of previous literature, we did not have specific hypotheses about relationships between the slow-γ frequency band activity and motor learning or SICI measures.

## 2 Methods

### 2.1 Experiment 1: MEG – motor learning – TMS

#### 2.1.1 Participants

In total, 33 individuals (age 24.9 years, range: 21–30 years, 14 male) gave their informed consent in accordance with Central University Research Ethics Committee approval (University of Oxford; MSD-IDREC-C2-2014-026 and MSD-IDREC-C1-2015-010) and the Declaration of Helsinki. All participants were right handed as assessed by the Edinburgh Handedness Inventory (Oldfield, 1971), had normal or corrected-to-normal vision, had no history of neurological or psychiatric disorders, had no metal implants, and reported no contraindications to TMS or MEG.

#### 2.1.2 Experimental design

**Fig. 1.**
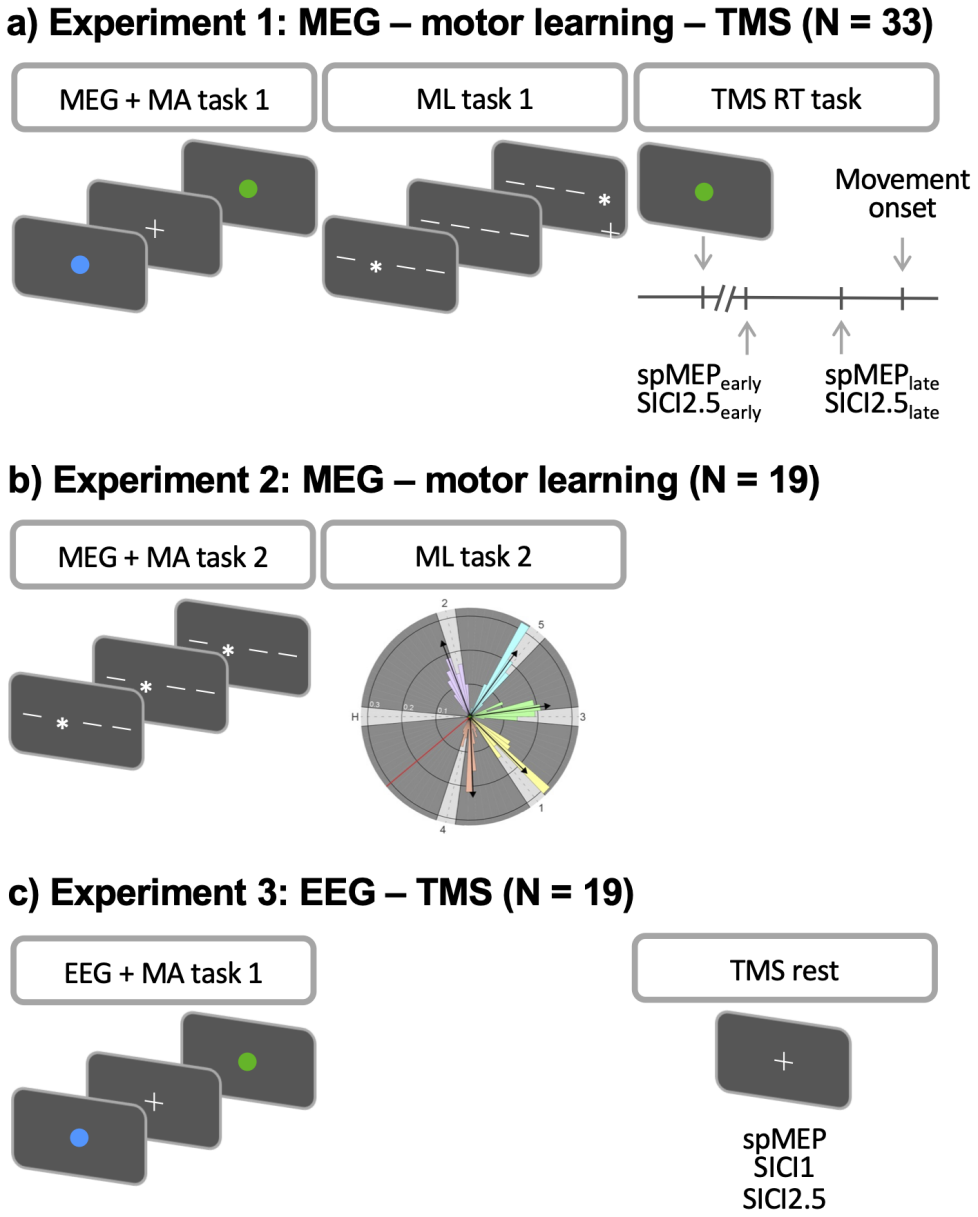
Experimental designs. (a) In experiment 1, participants performed a motor activation task (MA task 1) during MEG data acquisition, a motor learning task (ML task 1), and TMS. TMS measures (single-pulse MEP [spMEP] and short interval intracortical inhibition [SICI] with 2.5 ms interstimulus interval) were acquired during a simple reaction time task (RT task). During the RT task, spMEP and SICI were obtained at two different timings during movement preparation: an early time point (25% of mean RT, spMEP_early_, SICI_early_) and a late time point (65% of mean RT, spMEP_late_, SICI_late_). (b) In experiment 2, participants performed a motor activation task (MA task 2) during MEG data acquisition and a motor learning task (ML task 2). (c) In experiment 3, participants performed a motor activation task (MA task 1) during EEG data acquisition and TMS during rest with spMEP, SICI 1 ms, and SICI 2.5 ms.

All individuals completed a motor activation task (MA task 1) during EMG and MEG data acquisition, a motor learning task (ML task 1), and TMS on a single day (Fig. 1a).

#### 2.1.3 Motor activation task (MA task 1)

A Go/NoGo paradigm was used as MA task 1 to quantify β, slow-γ, and mid-γ activity. Specifically, a blue circle cue, presented for 200 ms, instructed participants to prepare for the abduction of the index finger of their right hand. The cue was then replaced by a fixation cross for 1 s (cue–stimulus interval). A subsequent visual stimulus presented for 200 ms (coloured circle: green for Go or red for NoGo) indicated whether they should perform (Go) or withhold (NoGo) the prepared motor response. Participants were instructed to respond as quickly as possible on the Go trials. The stimulus was then replaced by a fixation cross for a duration that varied randomly between 2 and 4 s (inter-trial interval). The task consisted of a total of 70 trials. NoGo trials (20% of all trials) were introduced to encourage participants’ attention to the task. Stimuli were generated using the MATLAB Psychophysics Toolbox version 3.0 package (Brainard, 1997).

#### 2.1.4 Motor learning task (ML task 1)

Motor learning was quantified via a visually cued motor sequence learning task (Nowak et al., 2017). Individuals were presented with four horizontal bars on a screen, each of which corresponded to a keyboard key. The bars were presented between 830 and 850 ms (jittered to prevent anticipation). When a bar changed into an asterisk, which was presented for 150 ms, individuals were instructed to press the corresponding key as quickly and accurately as possible. Blocks 2 to 14 (sequence blocks) comprised 3 repeats of a 10-item sequence constrained to a ratio of 3:3:2:2. Blocks 1 and 15 (random blocks) comprised 30 visual cues presented in a random order. Stimuli were generated using the MATLAB Psychophysics Toolbox version 3.0 package (Brainard, 1997).

#### 2.1.5 TMS during movement preparation

Intracortical inhibition was quantified during movement preparation using SICI with an interstimulus interval of 2.5 ms (Di Lazzaro et al., 2005;Kujirai et al., 1993;Ziemann et al., 1996). SICI during movement preparation has been interpreted as release of resting cortical inhibition (disinhibition) prior to the onset of a voluntary movement (Dupont-Hadwen et al., 2019;Reynolds & Ashby, 1999;Zaaroor et al., 2003). During the reaction time task, individuals were instructed to respond to a visual Go signal (green circle) by performing an index finger abduction of the right hand as quickly as possible. Visual stimuli appeared at random intervals (5–7 s) and the individuals were instructed to avoid anticipation of the Go signal and to relax their hands while the fixation cross was displayed on the screen. To assess the temporal specificity of GABAergic intracortical inhibition as assessed using SICI relative to movement onset, TMS measures were collected at two different times during movement preparation: early (25% of mean RT) and late (65% of mean RT). The 25% and 65% RT were adjusted to each individual’s mean RT collected from 20 trials without TMS (Hummel et al., 2009;Murase et al., 2004). Fifteen trials were obtained for each of the four pre-movement protocols, that is, single-pulse motor-evoked potentials (spMEP)_early_, spMEP_late_, SICI_early_, and SICI_late_, in a pseudorandomised order.

### 2.2 Experiment 2: MEG – motor learning

#### 2.2.1 Participants

In total, 19 individuals (age 23.7 years, range: 18–31 years, 9 females) gave their informed consent in accordance with Central University Research Ethics Committee approval (MSD-IDREC-C1-2014-053) and in accordance with the Declaration of Helsinki. All participants were right handed as assessed by the Edinburgh Handedness Inventory (Oldfield, 1971), had normal or corrected-to-normal vision, had no history of neurological or psychiatric disorders, had no metal implants, and reported no contraindications to MEG.

#### 2.2.2 Experimental design

All individuals completed a motor activation task (MA task 2) during MEG data acquisition and then performed a motor learning task (ML task 2,Fig. 1b).

#### 2.2.3 Motor activation task (MA task 2)

A phase-encoding paradigm (Mancini et al., 2012;Orlov et al., 2010;Sereno & Huang, 2006) was used as MA task 2 to quantify mid-γ activity. Specifically, continuous button presses of individual digits of the right hand (D2: index, D3: middle, D4: ring, D5: little) were performed on an MEG-compatible button box (Current Designs, Philadelphia, USA) with no rest periods. Participants were presented with four white circles, corresponding to the four digits. Each circle flashed eight times, at a rate of 1 Hz. The forward version of the task cycled from D2 to D5 inclusive, with the resulting 32 cycles repeated 8 times. The backward version of the task was identical in duration but cycled from D5 to D2 inclusive. The two versions were pseudorandomised across participants. Stimuli were created and presented using Neurobehavioral Systems Presentation software. The paradigm has been applied and validated using fMRI previously (Kolasinski et al., 2016,^2017^).

#### 2.2.4 Motor learning task (ML task 2)

Motor learning was quantified using a visuomotor learning task. Participants saw a dark grey circle with six light-grey 10° wide segments (home: 175–185°, gate 1: 304–314°, gate 2: 98–108°, gate 3: 355–5°, gate 4: 252–262°, gate 5: 46–56°) and a red cursor (Supplementary Fig. S1). Participants held a force transducer (Current Designs, Philadelphia, USA) in their right hand, resting on a pillow on their lap. Squeezing the force transducer moved the cursor anti-clockwise, while relaxing caused the cursor to move clockwise. The goal of the task was to move the cursor quickly and accurately between the start/end position (Home) and a sequential order of gates (home - gate 1 - home - gate 2 - home - gate 3 - home - gate 4 - home - gate 5 - home) by modulating the force exerted onto the transducer. All participants performed 24 min of repeated motor task practice. Stimuli were created and presented using Neurobehavioral Systems Presentation software.

### 2.3 Experiment 3: EEG – TMS

#### 2.3.1 Participants

In total, 18 individuals (age 24.8 years, range: 19–35, 7 female) gave their informed consent in accordance with Central University Research Ethics Committee approval (University of Oxford; MSD-IDREC-R81071-RE0001) and the Declaration of Helsinki. All participants were right handed as assessed by the Edinburgh Handedness Inventory (Oldfield, 1971), had normal or corrected-to-normal vision, had no history of neurological or psychiatric disorders, had no metal implants, and reported no contraindications to TMS or EEG. TMS data were not collected in 3 subjects due to high motor thresholds (>80% of the stimulator output at rest) resulting in 15 complete datasets.

#### 2.3.2 Experimental design

All individuals completed TMS and thereafter a motor activation task (MA task 1) during EMG and EEG data acquisition (Fig. 1c).

#### 2.3.3 Motor activation task (MA task 1)

To quantify low-γ, a version of the MA task 1 (used in experiment 1) was used. Only Go-trials were used (N = 140) with an inter-trial interval of 2.7 to 4.7 s, in steps of 0.5 s.

#### 2.3.4 TMS protocol

Intracortical inhibition was quantified during rest using SICI with an interstimulus interval of 1 and 2.5 ms. Specifically, spMEP, SICI 1 ms, and SICI 2.5 ms were measured in pseudorandomised order with 13 trials per condition, due to time constraints.

### 2.4 Data acquisition methods

#### 2.4.1 MEG data

MEG data were acquired with a whole-head 306-channel Elekta Neuromag system (204 planar gradiometers, 102 magnetometers). Data were sampled at 1,000 Hz with a band-pass filter of 0.03–330 Hz. Head position was continuously monitored with respect to the MEG sensors using four Head Position Indicator coils (HPI). The locations of HPI coils and three anatomical fiducials (the nasion and two preauricular points) were digitised using a 3D tracking system (Polhemus, Fastrak 3D) to define the subject-specific Cartesian head coordinate system. In addition, vertical and horizontal electrooculogram electrodes were used to allow for the detection and removal of eye-blink artefacts. MEG data were sampled at 1,000 Hz using a 0.03–330 Hz band-pass filter during digitalisation. Stimuli were back projected (Panasonic PT D7700E, Panasonic, Osaka, Japan) on a 43 x 54.5 cm screen placed 120 cm in front of the participant, with a spatial resolution of 1,280 x 1,024 and a refresh rate of 60 Hz.

#### 2.4.2 EEG data

EEG data were recorded with sintered Ag/AgCl electrodes and a 32-channel TMSi-Porti amplifier (TMS International, The Netherlands). Data were acquired with an amplitude resolution of 0.0715 μV and a sampling rate of 2,048 Hz. The EEG data were collected through an EEG cap from 12 electrodes placed on a subset of the 10/20 system with an increased resolution over the region of the left primary motor cortex (i.e., FC5, FC3, FC1, FCz, C5, C3, C1, CP5, CP3, CP1, Cz, and CPz). The ground Ag/AgCl electrode was placed on the left forearm. The impedance was kept below 30 kΩ for all electrodes during the recordings.

#### 2.4.3 TMS data

All TMS data were acquired using a monophasic BiStim TMS unit connected to a 70 mm figure-of-eight coil (Magstim Company Ltd). The left M1 FDI motor hotspot, that is, the position where spMEPs could be elicited in the right FDI muscle at the lowest stimulator intensity, was targeted. The TMS coil was held at 45° to the midsagittal line with the handle pointing posteriorly, resulting in anterior-posterior current direction. The hotspot was marked on a tight-fitting cap to ensure reproducible coil positioning. First, the 1 mV resting motor threshold (1 mV-MT) and active motor threshold (aMT) were determined. 1 mV-MT was defined as the stimulus intensity required to elicit spMEPs of a mean peak-to-peak amplitude of 1 mV over 10 trials in the relaxed FDI muscle. aMT was defined as the minimum stimulus intensity necessary to evoke spMEPs of ~0.2 mV peak-to-peak amplitude in at least 5/10 trials while individuals maintained ~30% of the maximum voluntary contraction of the FDI. For SICI, the conditioning stimulus was set at 70% of aMT and the test stimulus at 1 mV-MT.

#### 2.4.4 Surface EMG

Surface EMG was recorded during MA tasks 1 and 2, as well as during TMS from the FDI of the right hand using a belly-tendon montage with a ground electrode over the ulnar styloid process. During MEG, EMG data were simultaneously sampled at 1 kHz. During EEG and TMS, EMG data were sampled at 5 kHz, amplified, filtered (10–1,000 Hz), and recorded using a CED 1902 amplifier, a CED micro1401 A/D converter, and Signal software version 3.13 (Cambridge Electronic Design).

### 2.5 Data analysis

#### 2.5.1 MEG data analysis

External noise was reduced from MEG data using spatio-temporal signal–space separation (TSSS) and head movements (detected using HPI coils) corrected, both using MaxMove software as implemented in MaxFilter (Elekta Neromag, Elekta, Stockholm, Sweden; version 2.1). Further MEG data analyses were performed using the in-house OHBA Software Library (OSL:https://ohba-analysis.github.io/osl-docs/; version 2.2.0). Registration between a structural MRI template, that is, MNI152 standard-space T1-weighted average structural template image, and MEG data was performed with RHINO (Registration of Headshapes Including Nose in OSL) using nose and fiducial landmarks for co-registration and a single shell as forward model.

Continuous data were downsampled to 500 Hz. Further, a band-pass filter (1–245 Hz) and several notch filters were applied (49–55, 99–101, 149–151, 199–201 Hz). A wider notch filter around 50 Hz was used to suppress 50 Hz line noise, and a 53 Hz artefact present in this dataset was caused by the HPI coils. Time segments containing artefacts were identified by using the generalised extreme studentised deviate method (GESD,Rosner, 1983) at a significance level of 0.05 with a maximum number of outliers limited to 20% of the data on the standard deviation of the signal across all sensors in 1 s non-overlapping windows. The windows corresponding to outliers were excluded from all further analyses. Further denoising was applied using independent component analysis (ICA) using temporal FastICA across sensors (Hyvarinen, 1999). In total, 62 independent components were estimated and components representing stereotypical artefacts such as eye blinks, eye movements, and electrical heartbeat activity were manually identified and regressed out of the data. Data then were filtered into three frequency bands (β 13–30 Hz, slow-γ 30–60 Hz, mid-γ 60–90 Hz) and the following processing steps were performed separately for the three frequency bands.

Magnetometers and Planar-Gradiometers were normalised by computing the eigenvalue decomposition across sensors within each coil type and dividing the data by the smallest eigenvalue within each (Woolrich et al., 2011). Data were projected onto an 8 mm grid in source space (resulting in 3,559 virtual sensors) using a Linearly Constrained Minimum Variance (LCMV) vector beamformer (Van Veen & Buckley, 1988;Woolrich et al., 2011). Beamformer weights were estimated across a volumetric 8 mm three-dimensional grid cast within the inner skull of the MNI152 brain. A covariance matrix was computed across the whole time course and was regularised to 50 dimensions using principal component analysis (PCA) rank reduction (Quinn et al., 2018).

Epochs were defined relative to the movement onset (movement offset for β ERS) as identified by surface EMG. To identify movement onset and offset, EMG data were first high-pass filtered at 10 Hz. EMG data were then segmented from -1 to 3 s relative to the Go stimuli, and the envelope (root mean square, window = 80 ms) was computed. Using a non-overlapping moving standard deviation (movement onset: window = 24 ms, direction = forwards; movement offset: window = 120 ms, direction = backwards), movement onset and offset were defined as the first window exceeding the threshold (three standard deviations of the EMG activity between -600 and -200 ms relative to Go stimuli). Trials were excluded when the envelope, the reaction time (i.e., the time between Go stimuli and movement onset), or the movement time (i.e., the time between movement onset and offset) was identified as outliers using GESD at a significance level of 0.05. This approach resulted in 45.59 (SD = 4.88) out of 56 epochs per individual. MEG data were segmented from -2 to 2 s relative to movement onset (movement offset for β ERS).

Time–frequency analysis was applied to single trials and virtual sensors using a dpss-based multitaper (window = 1.6 s, steps = 200 ms) with a frequency resolution of 1 Hz. Segments were baseline corrected (-1 to -0.5 s, [-1.5 to -1 s for β ERS]) using the mean baseline across all trials. This procedure results in a time–frequency decomposition for each trial and each of the 3,559 virtual sensors. To detect the individual power, peak frequency, and peak voxel within each frequency band, only the virtual sensors within M1, following the Desikan–Killiany atlas (N = 92), were considered. For each trial, power was averaged across time, that is, from movement onset to movement offset (from movement offset to movement offset + 1 s for β ERS). This reduces four-dimensional power data (time, frequency, space, trial) to three-dimensional power data (frequency, space, trial). Further, three-dimensional power data were averaged across trials resulting in two-dimensional power data (frequency, space). The maximum (minimum for β ERD) of this two-dimensional power data was defined as an individual’s power, and defined the individual’s peak frequency and virtual sensor (Supplementary Fig. S2).

To illustrate the spatial properties of movement-related responses, we computed the movement-related power (as above, first averaging across time, i.e., from movement onset to movement offset [from movement offset to movement offset + 1 s for β ERS] and then averaged across trials) at the individual’s peak frequency for each of the 3,559 virtual sensors.

#### 2.5.2 EEG data analysis

EEG data were analysed using EEGLAB (2021.1) and Fieldtrip as released in SPM12. EEG data analysis resembled MEG data analysis as closely as possible. Data were downsampled to 250 Hz. A band-pass (1–95 Hz) and notch (49–51 Hz) filter were applied. Time segments containing artefacts were identified by using the generalised extreme studentised deviate method (GESD,Rosner, 1983) at a significance level of 0.05 with a maximum number of outliers limited to 20% of the data on the standard deviation of the signal across all sensors in 1 s non-overlapping windows. The windows corresponding to outliers were excluded from all further analyses. Data then were filtered into the frequency band of interest (i.e., slow-γ 30–60 Hz). Epochs were defined relative to the movement onset as identified by surface EMG using the same approach as outlined in the section*MEG data analysis*. EEG data were segmented from -2 to 2 s relative to movement onset. Time–frequency analysis was applied to single trials and channels using a dpss-based multitaper (window = 1.6 s, steps = 200 ms) with a frequency resolution of 1 Hz. Segments were baseline corrected (-1 to -0.5 s) using the mean baseline across all trials. Trial-wise movement-related power was obtained by averaging across time, that is, from movement onset to movement offset and then averaged across trials. The maximum of the resulting two-dimensional matrix containing the power between movement onset and offset across trials at each frequency within the frequency band at each channel was defined as an individual’s power and defined the individual’s peak frequency and channel.

#### 2.5.3 Motor learning task (ML task 1)

First individual RTs (i.e., time from cue onset to the correct button press) that were anticipatory (i.e., those that occurred before the cue) or outliers (i.e., RTs outside of the mean value ± 2 SD per each block) were discarded. A motor learning score was calculated for each individual as a percentage change from the RT in the first sequence block (block 2) to blocks 10–14 when the learning plateaued (Stagg, Bachtiar, et al., 2011). Thus, more negative scores represent more motor learning. One individual was excluded from this analysis due to non-compliance with instructions.

#### 2.5.4 Motor learning task (ML task 2)

Movement time (MT; time from movement onset, i.e., initiation to visit Gate 1, to movement offset, i.e. arrival at Home following gate 5 visit) and accuracy (i.e., absolute angular difference between the centre of the gate and the reversal point of the cursor) were extracted from each trial as behavioural measures. The self-paced character of the task means that despite a comparable total movement time across individuals ($MT_{total}$= 19.70 min,*SE*= 24.88 s,Supplementary Fig. S3a,b) due to the inter-individual differences in the trial-wise movement time, the number of completed trials differed substantially across individuals. The total movement time is defined as the sum of all trial-wise movement times within one individual, that is,$MT_{total}=\sum_{i}^{N}MT\left(i\right)$, whereNdenotes the number of trials per individual. Thus, motor learning is measured over the course of movement time (or time on task) rather than the number of repetitions, please note that a comparison between the two showed similar results (Schoenfeld, 2021). To accurately describe practice-induced changes, trial-wise metrics (error, i.e., deviation from turning point; movement time, i.e., movement onset to movement offset) were divided into six bins of equal movement time within the individual (i.e.,$MT_{bin}=\frac{MTtotal}{B}$, whereBdenotes the number of bins,$MT_{bin}$= 3.28 min,*SE*= 4.15 s,Supplementary Fig. S3c). Finally, the difference between the first and last bin reflects the amount of practice-related change, that is, motor learning, whereby more negative scores reflect more learning.

#### 2.5.5 TMS data analysis

Trials were excluded if the test pulse alone failed to elicit a reliable MEP (amplitude <0.1 mV), if there was precontraction in the target FDI muscle (EMG amplitude >0.1 mV in the 80 ms preceding the pulse), or, for the pre-movement TMS measures, EMG onset coincided with TMS pulse, or no response was made. The peak-to-peak amplitude for each MEP was then calculated. Any MEPs outside of the mean value ± 2 SD for each condition for each block were excluded. Next, a single iteration of Grubbs’ test with a significance level of 0.05 was performed for each TMS condition separately and any significant outliers were excluded. Collectively, these rejection criteria resulted in the exclusion of <5 trials per individual in any condition. SICI was expressed as a ratio of the mean conditioned MEP amplitude to the mean unconditioned MEP amplitude. For the pre-movement data, the TMS measures were analysed separately for each pre-movement time point (25% and 65% RT).

### 2.6 Statistical analysis

All Frequentist statistics were conducted as implemented in SPSS version 25 (SPSS Inc, Chicago, IL, USA). As Bayesian inference allows multiple hypotheses to be tested and can calculate the probability that one hypothesis is true relative to another hypothesis, correlation analysis was performed using Bayesian inference (JASP, JASP Team 2019, version 0.11.1) with default priors after outlier removal. The Bayes factor (BF) is the ratio of the likelihood of one particular hypothesis to the likelihood of another. We categorise BFs based using the heuristic classification scheme for BF_10_(Lee & Wagenmakers, 2014, p.105; adjusted fromJeffreys, 1961). Thus, for example, BF_10_= 10–30 denotes strong evidence, BF_10_= 3–10 moderate, and BF_10_= 1–3 anecdotal evidence for H_1_, while BF_10_= 1/3–1 denotes anecdotal, BF_10_= 1/10–1/3 moderate, and BF_10_= 1/30–1/10 strong evidence for H_0_. Outliers were identified for each correlation separately by bootstrapping the Mahalanobis distance (Schwarzkopf et al., 2012). To statistically compare correlations, Fisher’s z-transformation was applied to each correlation coefficient, resulting in normally distributed values*r’*with standard errors*s_r’_*. The null hypotheses (*r’*_1_-*r’*_2_= 0) were tested in R(psych) (Revelle, 2015) using the Student*t*test. Reported*p*-values are 2-tailed.

## 3 Results

### 3.1 MEG reveals expected ERD and ERS in the β band: Experiment 1

**Fig. 2.**
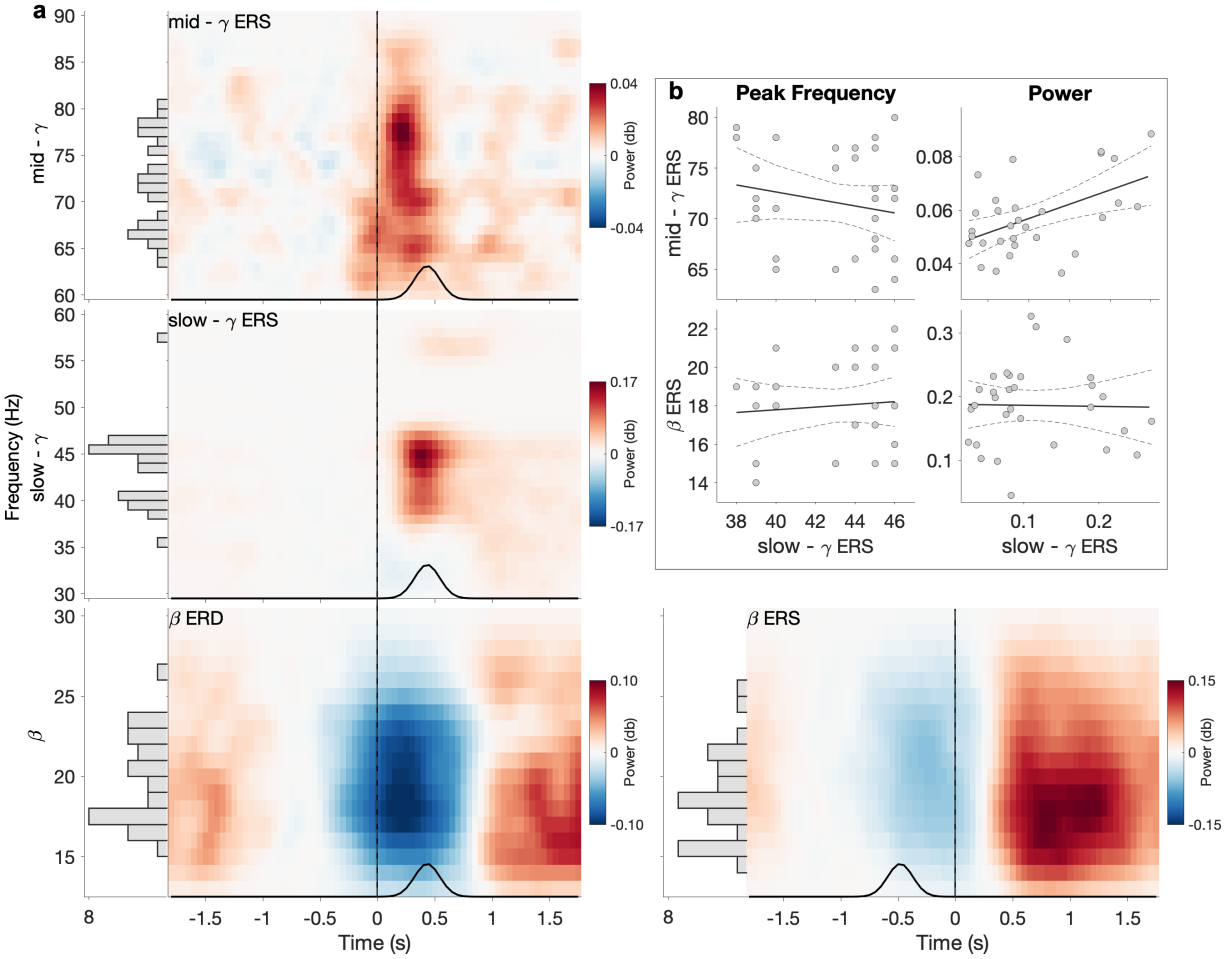
Temporal and spectral properties of movement-related responses from MEG data: Experiment 1. (a) Power for mid-γ (top), slow- γ (middle), β (bottom, left β ERD, right β ERS). Time–frequency plots show the power at the peak voxel within the left sensorimotor cortex obtained per subject and frequency bands separately, and then averaged across subjects (see section “MEG data analysis”). Data are locked to movement onset (movement offset for β ERS, as identified by EMG and highlighted by the black vertical line). The black line represents the distribution of movement offsets (movement onsets for β ERS). Power is shown relative to baseline (-1 to -0.5 s relative to movement onset [-1.5 to -1 s relative to movement offset for β ERS]). Side panel histograms illustrate the distribution of individual peak frequencies (bin size = 1 Hz). (b) Correlations for peak frequency (left) and power (right) between slow-γ ERS and β ERS (bottom) as well as between slow-γ ERS and mid-γ ERS (top). Power represents the trial-wise average from movement onset to movement offset for slow-γ and mid-γ (from movement offset to movement offset + 1 s for β ERS) at the individual’s peak voxel and at the individual’s peak frequency (see section “MEG data analysis”). Dashed lines represent the 95% confidence intervals.

MEG data from experiment 1 show clear movement-related changes in power in all three frequency bands (β, slow-γ, mid-γ), characterised by different spectral–temporal–spatial properties. Specifically, we observed a clear β ERD during movement and a β ERS after movement termination. In line with previous studies, the β ERD started before movement onset, plateaued between movement onset and movement offset, and terminated after movement offset (Fig. 2a, bottom left). The mean β ERD peak frequency was 19.33 Hz (range 15–26 Hz). There was moderate evidence for a lack of relationship between peak frequency and power (*r*= 0.011, BF_10_= 0.22) across individuals. Again, consistent with prior observations, the β ERS started after movement offset and lasted for roughly 1 s (Fig. 2a, bottom right). The mean β ERS peak was 18.21 Hz (range 14–25 Hz). There was anecdotal evidence for a lack of relationship between peak frequency and power (*r*= -0.284, BF_10_= 0.74).

### 3.2 Two distinct patterns of movement-related γ activity: Experiment 1

We then wanted to investigate movement-related activity in the γ bands of the same data. In the slow-γ band, we observed a strong slow-γ ERS, which started after movement onset, reached its peak at the time of movement offset, and decreased after movement offset, suggesting that the slow-γ ERS was temporally aligned with movement offset (Fig. 2a, centre left). The mean slow-γ peak frequency was 43.06 Hz (range 35–57 Hz), and moderate evidence for a lack of relationship between peak frequency and power was found (*r*= -0.034, BF_10_= 0.22). In the mid-γ band, we also observed an ERS. This mid-γ ERS started at movement onset, reached its peak between movement onset and movement offset, and terminated around movement offset (Fig. 2a, top left), therefore, showing a temporal alignment with movement, unlike the slow-γ ERS. The mean mid-γ peak frequency was at 71.36 Hz (range 63–80 Hz), and there was anecdotal evidence for a lack of relationship between peak frequency and power (*r*= 0.249, BF_10_= 0.55).

To our knowledge, while movement-related slow-γ has been reported previously (Crone et al., 1998;Szurhaj et al., 2006), its properties have not been fully characterised. We, therefore, sought to investigate whether this pattern of neural activity was distinct from the movement-related β ERS and mid-γ ERS. We performed four Bayesian pairwise correlations to test whether the peak frequency or power of the slow-γ ERS was related to these measures derived from the β ERS or mid-γ ERS (Fig. 2b). We found moderate evidence for a lack of relationship between slow-γ ERS and β ERS peak frequency (*r*= -0.196, BF_10_= 0.38) and anecdotal evidence for a lack of relationship between slow-γ ERS and mid-γ ERS peak frequency (*r*= 0.088, BF_10_= 0.26). In terms of power, there was moderate evidence for a lack of a relationship between slow-γ ERS and β ERS (*r*= -0.019, BF_10_= 0.22), but strong evidence for a relationship between slow-γ ERS and mid-γ ERS (*r*= 0.514, BF_10_= 12.61).

**Fig. 3.**
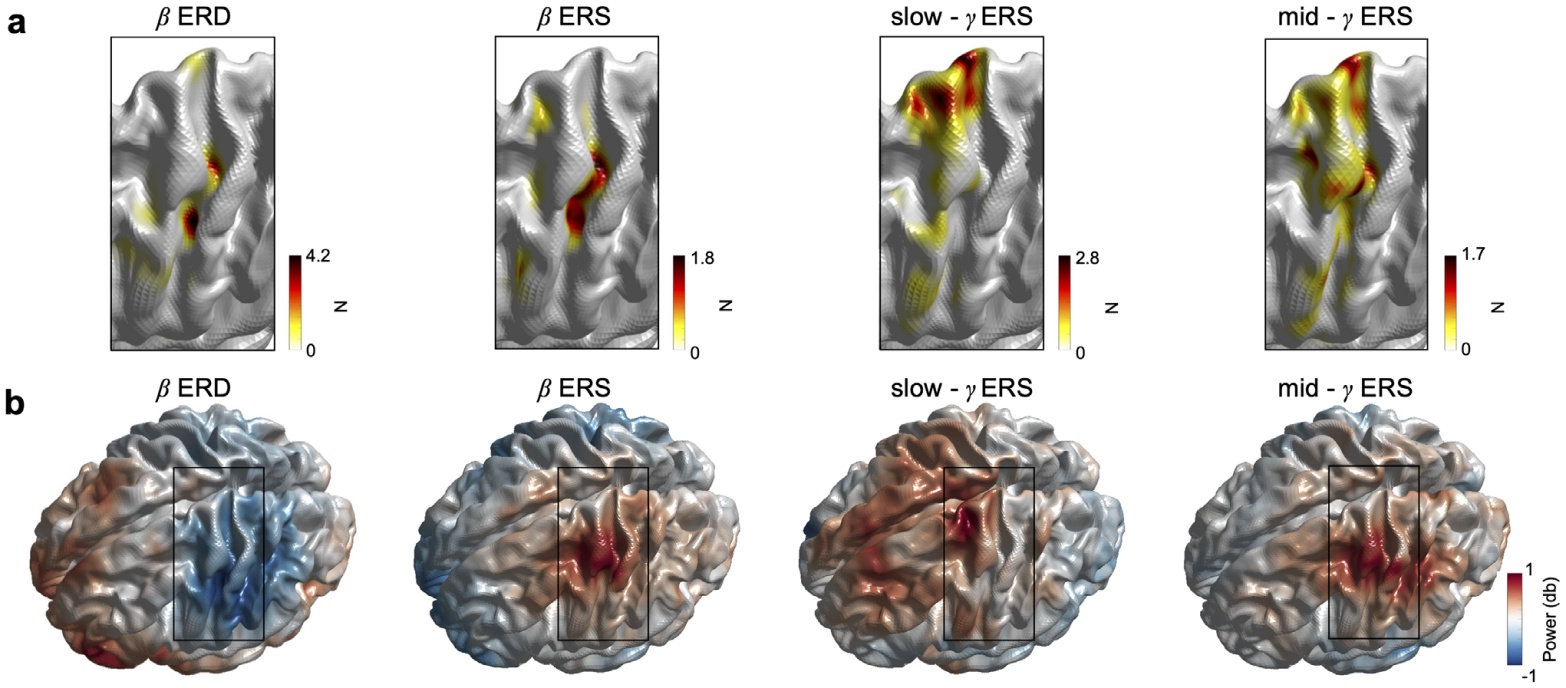
Spatial properties of movement-related responses from MEG data: Experiment 1. (a) Heatmap of the number of selected virtual sensors within the sensorimotor cortex separately for β ERD, β ERS, slow-γ, and mid-γ. For visualisation data are interpolated. (b) Power maps separately for β ERD, β ERS, slow-γ, and mid-γ. Power represents the trial-wise average from movement onset to movement offset for β ERD, slow-γ, and mid-γ (movement offset to movement offset + 1 s for β ERS) at each of the 3,559 virtual sensors at the individual’s peak frequency (see section “MEG data analysis”).

Next, we examined the spatial properties of the movement-related slow-γ ERS compared with the β ERD, β ERS, and mid-γ ERS. Therefore, we considered the group heatmaps of the peak virtual sensors and power maps. In line with previous findings, the group heatmaps of the peak virtual sensors for β ERD and β ERS were relatively focal with the hotspot posterior and relatively central on the superior-inferior axis within the sensorimotor cortex (Fig. 3a). In contrast, the hotspot for the slow-γ was more superior and central on the anterior-posterior axis. Finally, the heatmap for the mid-γ was less focal, encompassing the hotspot of the β and the slow-γ frequency range.

The spatial properties of β ERD, β ERS, mid-γ, and slow- γ were evaluated using the heatmaps of the selected virtual sensors within the left sensorimotor cortex (Fig. 3a) and the whole-brain power maps. The power maps are qualitatively comparable for β ERD, β ERS, and mid-γ, all with the local maxima within the left sensorimotor cortex. This is further confirmed by very localised heatmaps of the selected virtual voxel, especially for β ERD and β ERS. In contrast, the whole-brain power map shows a less localised pattern, which includes M1, but not S1, and extends more centrally and frontally. Comparing the locations of the individual virtual sensors yields significantly more dorsal locations within the sensorimotor cortex for slow-γ ERS (*M*= 61.33 mm,*SD*= 13.81 mm) compared with β ERS (*M*= 41.70 mm,*SD*= 11.96 mm;*p*< 0.001,*t*(32) = 5.38) and mid-γ ERS (*M*= 49.70 mm,*SD*= 16.70 mm;*p*= 0.004,*t*(32) = 3.06).

### 3.3 Slow-γ ERS peak frequency is related to individuals’ GABAergic intracortical inhibition during movement preparation: Experiment 1

**Fig. 4.**
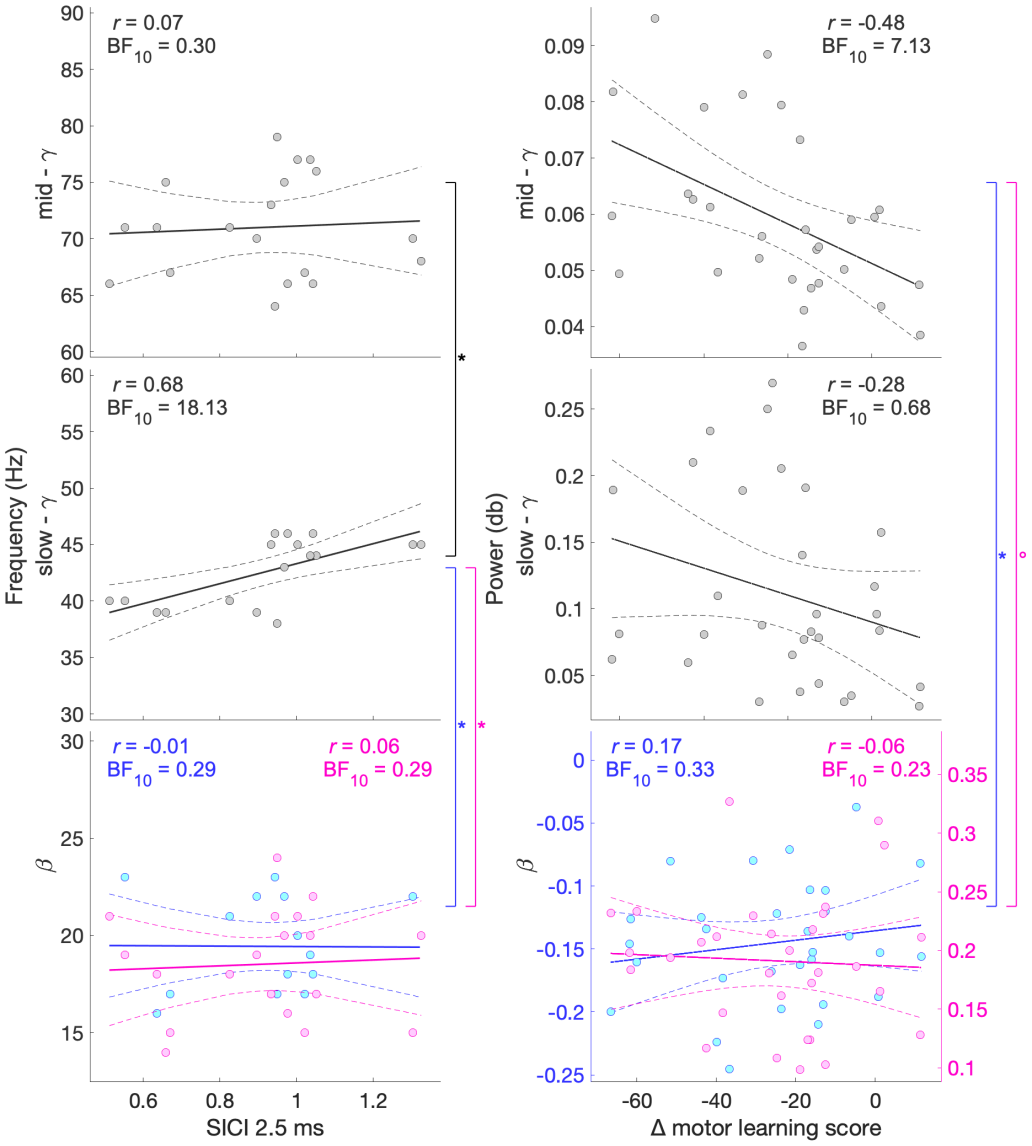
Relationship between MEG peak frequency and SICI (left) and MEG power and motor learning score (right) for β ERD (bottom blue), β ERS (bottom pink), slow-γ (middle), and mid-γ (top): Experiment 1. Power represents the trial-wise average from movement onset to movement offset for β ERD, slow-γ, and mid-γ (from movement offset to movement offset + 1 s for β ERS) at the individual’s peak voxel and at the individual’s peak frequency (see section “MEG data analysis”). Dashed lines represent the 95% confidence intervals. All data are from experiment 1. °*p*< 0.1, *p< 0.5.

Next, we investigated the neurophysiological underpinnings of the movement-related activity we observed in MEG data of experiment 1. We found that movement-related activity in the γ band is related to movement-related GABAergic intracortical inhibition, we found strong evidence for a positive relationship between pre-movement SICI amplitude and slow-γ peak frequency (*r*= 0.677, BF_10_= 18.13). There was moderate evidence for a lack of relationship between pre-movement SICI amplitude and peak frequency in other bands (β ERD:*r*= -0.010, BF_10_= 0.29; β ERS:*r*= 0.061, BF_10_= 0.29; mid-γ:*r*= 0.07, BF_10_= 0.295,Fig. 4, left). The observed relationship between pre-movement SICI and slow-γ peak frequency was significantly different from the relationships observed in other bands (SICI and slow-γ peak frequency vs. SICI and β ERD peak frequency:*z*= -2.26,*p*= 0.024; SICI and slow-γ peak frequency vs. SICI and β ERS peak frequency:*z*= -2.10,*p*= 0.036; SICI and slow-γ peak frequency vs. SICI and mid-γ peak frequency:*z*= -2.07,*p*= 0.039).

Having demonstrated a significant relationship between pre-movement SICI and slow-γ peak frequency, we then wished to explore the temporal specificity of this effect. Therefore, we investigated the relationship between pre-movement SICI and slow-γ peak frequency, for SICI_early_and SICI_late_separately, in post hoc analyses. There was anecdotal evidence for a positive relationship between slow-γ peak frequency and SICI early in movement preparation (SICI_early_,*r*= 0.433, BF_10_= 1.78) and strong evidence for a positive relationship between slow-γ peak frequency and SICI late in movement preparation (SICI_late_,*r*= 0.641, BF_10_= 10.41). There was no significant difference between these two correlations (*z*= -0.84,*p*= 0.401). There were no significant correlations between the peak frequency of any band and subsequent motor learning.

### 3.4 Mid-γ power correlates with subsequent motor learning: Experiment 1

Finally, we wished to investigate the behavioural importance of these movement-related signals.

Firstly, it was important to determine whether participants were able to learn the task. As expected, RT decreased (Supplementary Fig. S4). Statistically, we observed a significant reduction in RT (*F*_(14,336)_= 9.015;*p*< 0.001) between the first sequence block (block 2) and the later sequence blocks, when learning plateaued (blocks 10–14). In contrast, there was no significant difference in mean RT between the first random block (block 1) and the last random block (block 15;*t*_(32)_= 0.885;*p*= 0.383), whereas there was a significant difference between block 14 (the final learning block) and block 15 (the second random block) (*t*_(28)_= -6.899;*p*< 0.001), suggesting that improvements in RT occurred via learning of a specific sequence and not generic skill learning. There was also no significant difference between the RT from blocks 10 to 14, which were on the plateau of the learning curve (*F*_(4,108)_= 0.440;*p*= 0.780).

We found moderate evidence for a negative relationship between motor learning score and mid-γ power (*r*= -0.481, BF_10_= 7.13,Fig. 4, right), such that higher mid-γ power was related to greater motor learning. There was moderate evidence for a lack of relationship between motor learning score and power in other bands (β ERD:*r*= 0.166, BF_10_= 0.33; β ERS:*r*= -0.056, BF_10_= 0.23; slow-γ:*r*= -0.281, BF_10_= 0.68). The observed relationship between motor learning score and mid-γ power was different from the relationship between motor learning score and β ERD power (*z*= -2.55,*p*= 0.011) and β ERS power (*z*= -1.72,*p*= 0.085). There were no significant relationships between the power in any band and SICI.

### 3.5 Replication of correlation analyses: Experiment 2 and Experiment 3

Finally, we wished to replicate the relationships between SICI and slow-γ peak frequency as well as the relationship between motor learning and mid-γ power described above in independent datasets. Note that these constitute conceptual replications of the correlation analyses, rather than strict methodological replications. Experiments 2 and 3 were performed on a new cohort of participants than experiment 1. In addition, we used a different motor learning task in experiments 1 and 2, and a different motor activation task and recording modality (i.e., EEG instead of MEG) in experiments 1 and 3.

**Fig. 5.**
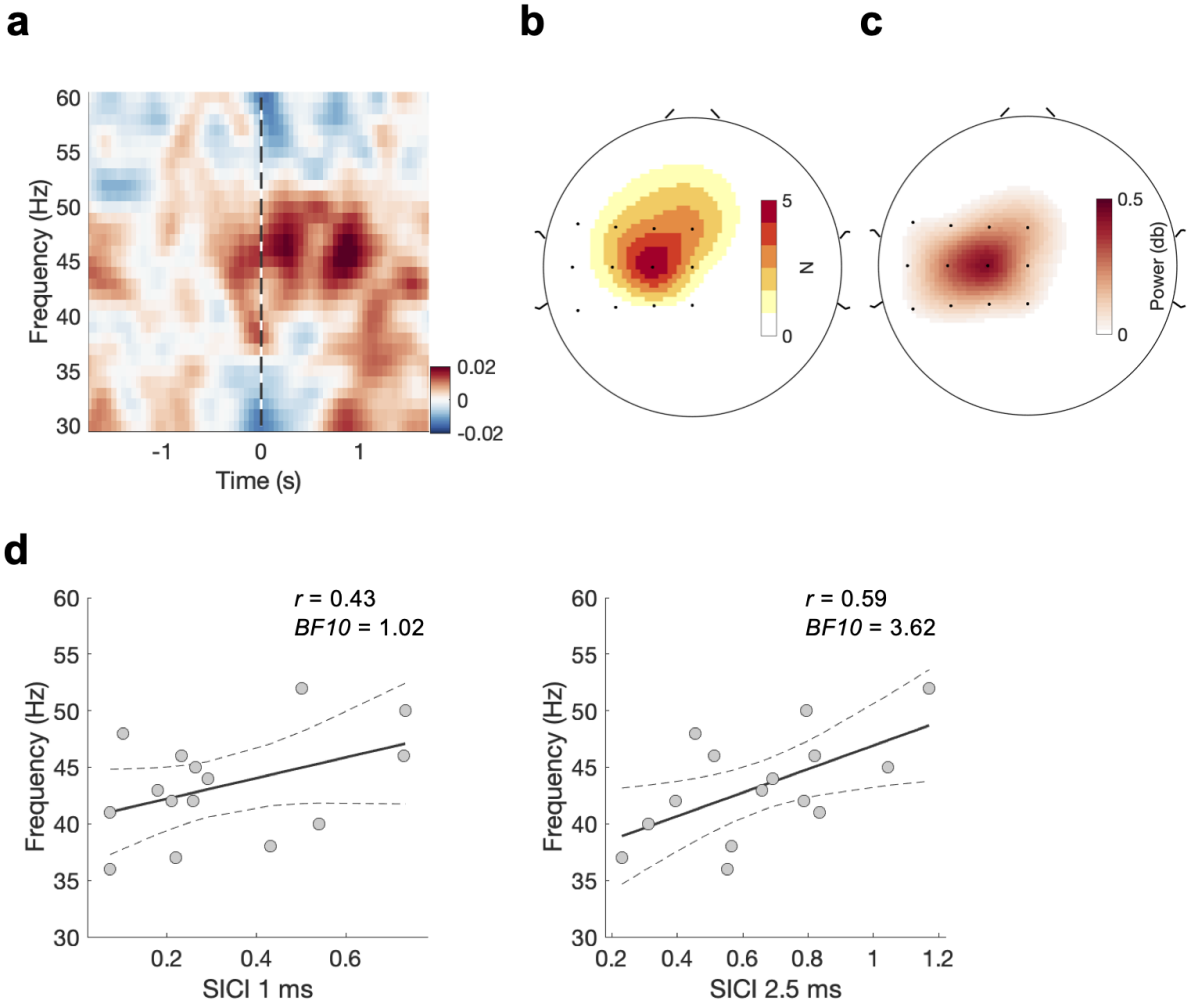
Replication of relationship between slow-γ (EEG) and SICI: Experiment 3. (a) Power from EEG data relative to baseline (-1 to -0.5 s relative to movement onset). Time–frequency plot shows the power at the peak channel within the EEG sensor array obtained per subject for the slow-γ frequency band, and then averaged across subjects (see section “EEG data analysis”). Data are locked to movement onset (as identified by EMG and highlighted by the black vertical line). (b) Heatmap of the number of selected EEG electrodes for slow-γ. For visualisation data are interpolated. (c) Power map slow-γ. Power represents the trial-wise average from movement onset to movement offset at each of the 12 EEG sensors at the individual’s slow-γ peak frequency (see section “EEG data analysis”). (d) Relationship between EEG-derived slow-γ peak frequency and SICI (left SICI 1 ms, right SICI 2.5 ms). Dashed lines represent the 95% confidence intervals. °*p*< 0.1, *p< 0.5.

Figure 5ashows the power across all subjects in the slow-γ at the EEG sensor with the strongest response in the slow-γ frequency range. Like experiment 1, albeit not as clearly defined, a significant slow-γ ERS can be observed. Again, the slow-γ ERS peaks after the movement onset and the power map (Fig. 5b), as well as the heatmap of the selected sensor (Fig. 5c), have their maxima more central and frontal, compared with the EEG site C3. In line with the relationship described in experiment 1, using EEG data and SICI (experiment 3; SICI 1 ms, SICI 2.5 ms) we found no evidence for a positive relationship between SICI 1 ms and slow-γ peak frequency (*r*= 0.428, BF_10_= 1.02) and moderate evidence for a positive relationship between SICI 2.5 ms and slow-γ peak frequency (*r*= 0.588, BF_10_= 3.617,Fig. 5d). The two relationships were not significantly different from each other (*z*= -0.41,*p*= 0.682).

**Fig. 6.**
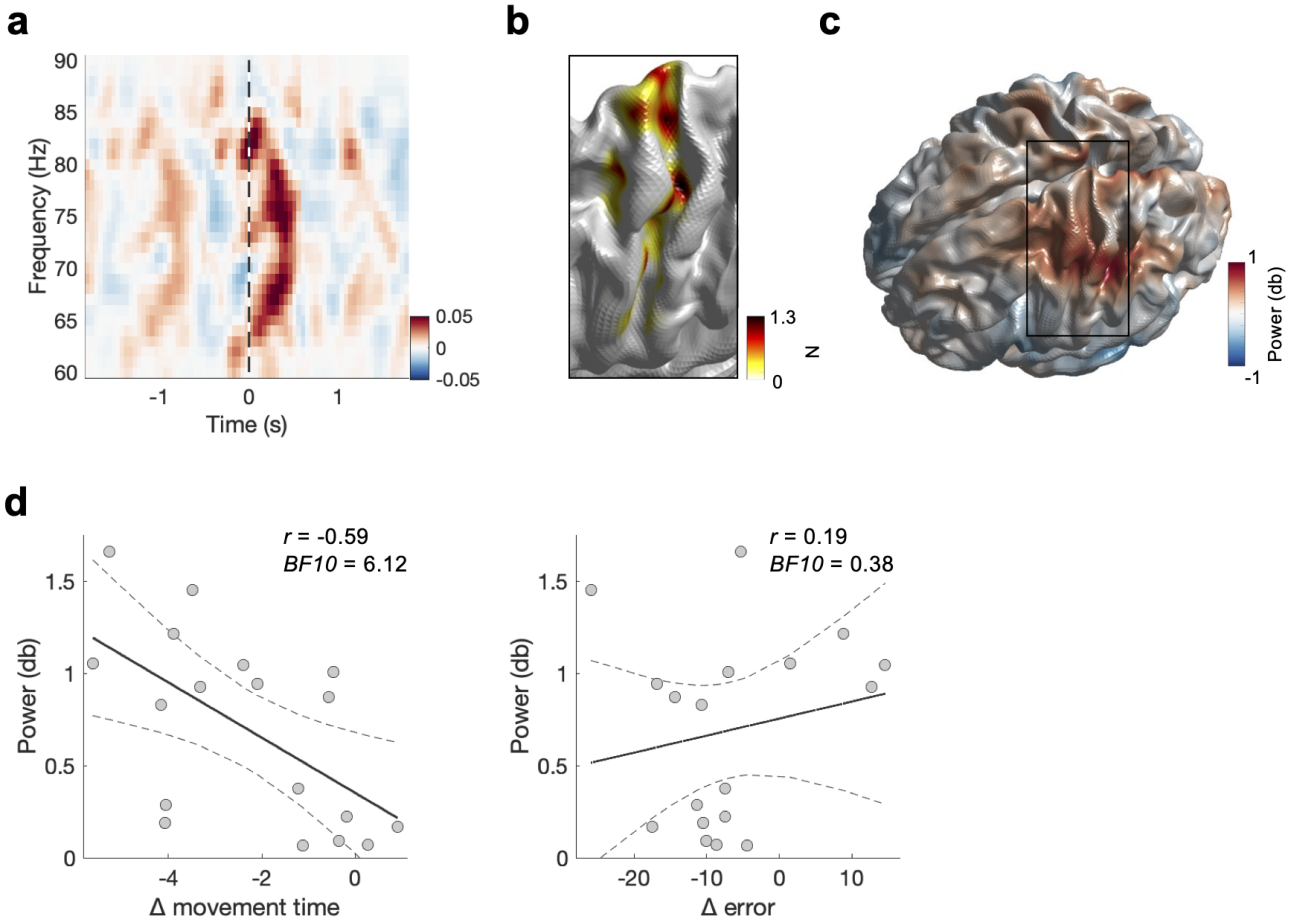
Replication of relationship between mid-γ (MEG) and motor learning (ML task 2): Experiment 2. (a) Power from MEG data relative to baseline (-1 to -0.5 s relative to movement). Time–frequency plot shows the power at the peak voxel within the left the sensorimotor cortex obtained per subject for the mid-γ frequency band, and then averaged across subjects (see section “MEG data analysis”). Data are locked to button presses (as identified by EMG and highlighted by the black vertical line). (b) Heatmap of the number of selected virtual sensors within the sensorimotor cortex mid-γ. For visualisation data are interpolated. (c) Power map for mid-γ. Power represents the trial-wise average from movement onset to movement offset at each of the 3,559 virtual sensors at the individual’s mid-γ peak frequency (see section “MEG data analysis”). (d) Relationship between MEG-derived mid-γ power motor learning score (left movement time, right error). Power represents the trial-wise average from movement onset to movement offset for slow-γ and mid-γ (from movement offset to movement offset + 1 s for β ERS) at the individual’s peak voxel and at the individual’s peak frequency (see section “MEG data analysis”). Dashed lines represent the 95% confidence intervals. °*p*< 0.1, *p< 0.5.

Lastly, we used an independent dataset (experiment 2) to replicate the relationship between mid-γ and motor learning described above.Figure 6ashows the power across all subjects in the mid-γ at the virtual MEG sensor with the strongest response in the mid-γ frequency range. Like experiment 1, a significant mid-γ ERS can be observed after the movement onset. Note that the rate of the movement was 1 Hz, resulting in a repeating mid-γ ERS pattern. As in experiment 1, the spatial distribution of the mid-γ ERS power shows a clear hotspot over the left hand area within the sensorimotor cortex (Fig. 6c). Next, we examined whether participants learned this motor task. Practice-related changes in movement time and error were analysed using two one-way repeated-measured analyses of variance (ANOVA) with bins (1, 2, 3, 4, 5, 6) as within-subjects factor. We found that practice led to faster (reduced movement time at constant inter-gate intervals,*N*= 19,*F_5,90_*= 7.35,*p*< 0.001,$\eta_{p}^{2}$= 0.290) and more accurate (reduced deviation from target,*F_5,90_*= 2.89,*p*= 0.018,$\eta_{p}^{2}$= 0.138) execution of the task (Supplementary Fig. S1c). Correlation analyses revealed moderate evidence for a negative relationship between practice-related change in movement time and mid-γ power (*r*= -0.587, BF_10_= 6.12,Fig. 6b), but anecdotal evidence for a lack of relationship between practice-related change in error and mid-γ power (*r*= 0.189, BF_10_= 0.379,Fig. 6b).

## 4 Discussion

This work aimed to examine the physiological basis and functional significance of movement-related γ activity in the sensorimotor cortex. We identified two distinct patterns of movement-related*y*activity in the sensorimotor cortex, characterised by different temporal–spectral–spatial properties. We went on to investigate the physiological correlates of these and identified a correlation between sensorimotor slow-γ peak frequency and SICI amplitude in M1, such that individuals with a higher slow-γ peak frequency showed less GABAergic intracortical inhibition. Finally, in line with previous work, we found that a higher sensorimotor mid-γ power was related to better individual performance in a motor learning task.

### 4.1 Two distinct movement-related patterns of γ activity

Animal studies and human direct cortical recordings have suggested the presence of two distinct patterns of activity within the γ band in M1, but until now it has proved difficult to robustly separate them with transcranial approaches. By optimising our MEG analysis, that is, separate beamformer for each sub-band and high-precision peak frequency/spatial location estimation, we have demonstrated the presence of two movement-related γ activity patterns within the sensorimotor cortex.

Given the paucity of previous transcranial studies focussing on slow-γ movement-related activity, we first examined whether the slow-γ activity seen here represented a distinct neural activity pattern or was merely an extension of either post-movement β ERS or movement mid-γ ERS. We then reasoned that if the slow-γ ERS reflected activity within the same local microcircuits as either the post-movement β ERS or movement mid-γ ERS, we would expect to observe systematic relationships on a subject-by-subject basis between slow-γ and β or mid-γ. We only found a relationship between slow-γ ERS power and mid-γ ERS power, but note that inter-individual differences in MEG power can also be explained by other factors, such as head size (Quinn et al., 2024). The absence of other relationships adds weight to the hypothesis that the slow-γ ERS is a distinct movement-related activity pattern but is in itself not conclusive. We, therefore, went on to investigate both the temporal and spatial domain of the slow-γ ERS, demonstrating that it is dissociable from both the post-movement β ERS and movement mid-γ ERS in both domains. Slow-γ ERS appeared to be temporally aligned with the movement offset, rather than movement onset, like the mid-γ ERS, or post-movement, like β ERS. Moreover, the spatial distribution of the slow-γ ERS is more frontal and central than the spatial distribution of the mid-γ ERS and the β ERS, both localised to the sensorimotor cortex. This could indicate that movement-related slow-γ is generated by a more complex set of sources, which include motor, premotor, and frontal sites. The central shift is in line with ECoG data (Crone et al., 1998) and might reflect a role in motor programming as well as activation. The frontal component might reflect a separate movement-related source or muscle activity. However, in EEG, the amount of muscular contamination seems to be lower around 40 Hz, compared with 80 Hz (Whitham et al., 2007,^2008^). As we do not see a similar frontal component in the mid-γ, we cautiously believe that the frontal component slow-γ does not reflect muscle activity. High signal-to-noise MEG recordings allowing ultra-high spatial resolution analysis (similar toZich et al., 2023) are needed to further elaborate the spatial origins of the slow-γ ERS.

Taken together, the data suggest that two distinct patterns of movement-related γ activity are seen in the human sensorimotor cortex. The next question we aimed to address is the likely cellular basis of these two distinct γ activity patterns.

### 4.2 Slow-γ activity may arise from superficial cortical layers

A commonly held hypothesis states that activity in the lower cortical layers is predominantly slower than that in the more superficial layers (i.e., frequency–layer gradient), suggesting different functional roles of superficial and deep layers. This is supported by animal studies in primary sensory areas (e.g.,Buffalo et al., 2011;Haegens et al., 2015;Roopun et al., 2006;Spaak et al., 2012). Moreover, human laminar MEG showed that visual α activity and sensorimotor β activity localise more towards the white matter surface approximating infragranular origin than to the pial surface, while visual and sensorimotor γ activity localise more towards the pial surface approximating supragranular origin than to the white matter surface (Bonaiuto et al., 2018). However, there is also evidence challenging the frequency–layer gradient by suggesting deeper cortical layers as the origin for γ activity. For example, in the visual cortex of behaving mice, γ activity has been linked to parvalbumin (PV)-positive GABAergic interneurons (Chen et al., 2017), which are most densely populated in layer V (Fagiolini et al., 2004;Sohal et al., 2009). Further, auditory in vitro work revealed two distinct γ activities, 30–45 and 50–80 Hz, localised to layer II/III and layer IV, respectively (Ainsworth et al., 2011). The precise neural basis of γ activity in the primary M1, as opposed to primary sensory regions, has yet to be determined (Whittington et al., 2011). Translating the findings directly from studies performed in the sensory areas to M1 must be done with care, as the circuit organisation of M1 differs fundamentally from that of sensory areas (Shipp, 2005;Shipp et al., 2013;Yamawaki et al., 2014).

Here we tested the relationship between β, slow-γ and mid-γ, and SICI amplitude, a direct measure of local GABAergic intracortical inhibition, quantified via TMS. TMS preferentially stimulates more superficial neurons, particularly at the intensities used here (Siebner et al., 2009). Further, computational modelling studies have demonstrated that TMS effects can be explained by activity within the canonical microcircuit, which includes layer II/III and layer V excitatory pyramidal cells, inhibitory interneurons, and cortico-cortical and thalamo-cortical inputs (Di Lazzaro & Ziemann, 2013). We found a specific relationship between local GABAergic intracortical inhibition and slow-γ activity, which was not observed for either the β or mid-γ activity.

In summary, the data presented here may suggest that movement-related slow-γ activity arises from neuronal circuits containing layer II/III interneurons. The functional role of movement-related slow-γ activity is less clear. Previous work has postulated that it may directly reflect motor output (Crone et al., 1998).

### 4.3 Mid-γ activity may reflect activity in learning-related microcircuits

In light of the significant correlation between slow-γ and SICI amplitude, the absence of the same relationship for mid-γ could be interpreted in at least two ways. First, in human M1 slow-γ and mid-γ stem both from superficial layers, but from different populations or microcircuits, with the one underlying slow-γ, but not mid-γ, being GABAergic as measured using SICI. This would be in line with the frequency–layer gradient reported in sensory areas (Bonaiuto et al., 2018;Buffalo et al., 2011;Haegens et al., 2015;Roopun et al., 2006;Spaak et al., 2012).

Second, while slow-γ arises superficially, mid-γ arises from deeper layers, such as layer V. This is in conformity with other animal work in sensory areas (Ainsworth et al., 2011;Chen et al., 2017;Sohal et al., 2009) and supported by the functional role of M1 mid-γ. We demonstrated that the power of mid-γ activity elicited by a simple movement predicted the ability to learn a motor skill on a subject-by-subject basis. This result could be replicated using an independent dataset comprising a different motor activation task and a different motor learning task. Regarding the movement activation tasks, as movement-related mid-γ is very brief (Cheyne & Ferrari, 2013), the γ response to single movements (MA task 1) and 1 Hz paced movements (MA task 2) is not expected to be significantly different (Muthukumaraswamy, 2010). Regarding the motor learning tasks, experiment 1 quantifies motor learning using RT differences in a visually cued motor sequence learning task, while in the conceptual replication, uses a visuomotor learning task and quantified error and movement time. While comparisons across different motor tasks are sparse, the pathway from goal to action is similar across different motor learning tasks (Krakauer et al., 2019), which is why we believe that it is reasonable to use different motor learning tasks across different experiments. In turn, it could enhance generalisability and specificity of the effect. Here, we found that mid-γ relates to learning-related changes in reaction time (experiment 1) and learning-related changes in movement time (experiment 2), but not learning-related changes in error (experiment 2), indicating a role of mid-γ for faster movements. The result is further in line with previous work. For example, we have shown that an individual’s response to 75 Hz tACS relates to his or her ability to learn a visually cued motor sequence learning task (Nowak et al., 2017), and further, when amplitude modulated by an underlying theta pattern, improves acceleration of a ballistic thumb abduction in healthy adults (Akkad et al., 2021). The finding of a specific relationship between mid-γ activity and plasticity is consistent with data from animal recordings, suggesting that microcircuits containing α-1 GABA-A synapses, predominantly found in the PV-rich layer V in M1, are a major neural substrate for plasticity, at least in the visual cortex (Fagiolini et al., 2004). Together, the origin of mid-γ activity is not completely understood, but mid-γ activity seems to play a role in motor learning.

### 4.4 Limitations

All experiments used non-invasive recordings to indirectly study changes in movement-related activity in the motor cortex. While this approach provides an unrivalled ability to understand activity in the healthy human system, it has inherent limitations in terms of the conclusions we can draw. Specifically, here, it was difficult to accurately quantify activity around 50 Hz due to power line noise. In addition, due to an artefact caused by the HPI coils at 53 and 54 Hz, we had to widen the standardly employed line noise notch filter to 49–55 Hz for the MEG data. This had direct implications on our assessment of the peak frequency of slow-γ ERS in experiment 1. In experiment 3, we used EEG to quantify γ activity. This can be difficult to interpret as it carries the risk of contamination from overlying muscles. However, this activity is usually more prominent in the mid-γ compared with slow-γ band (Whitham et al., 2007,^2008^), and has a broad spectral width (for review see (Muthukumaraswamy, 2013). As the frequency of the slow-γ ERS is lower than what has been reported for muscle activity and a comparable pattern was observed using MEG, we believe that this EEG activity reflects primarily neural activity.

### 4.5 Conclusions

The findings presented here allow us to create a theoretical framework for γ activity in the human sensorimotor cortex, as follows: there are two patterns of movement-related γ activity in the human motor cortex (slow-γ and mid-γ), with differential temporal, spectral, and spatial properties. The frequency of movement-related slow-γ activity is related to GABAergic intracortical inhibition but does not play a direct role in motor plasticity*in vivo*. One possibility is that slow-γ arises from GABA-A microcircuits in layer II/III. Mid-γ activity predicts motor learning, but whether it originates from layer II/III or V is not yet clear. This framework draws together findings from this paper and the literature and provides several hypotheses that can be directly tested.

## Supplementary Material

Supplementary Material

## Data Availability

We will consider requests to access the raw data in a trusted research environment as part of a collaboration. Contact:catharina.zich@ndcn.ox.ac.uk.
