# Supplementary material for: Human motor cortical gamma activity relates to GABAergic intracortical inhibition and motor learning
Source: Imaging Neurosci (Camb). 2025 Apr 24;3:imag_a_00538. doi: 10.1162/imag_a_00538 (PMC12319822; doi:10.1162/imag_a_00538)
Supplement: Supplementary Material [file imag_a_00538-supp.pdf]

Supplementary Information

Human motor cortical gamma activity relates to GABAergic intracortical inhibition and motor learning

Catharina Zich*, Magdalena Nowak*, Emily L Hinson, Camille Lasbareilles, Valentina Mancini, Alek Pogosyan, Oana Puicar, Ioana-Florentina Grigoras, Patricia Cambalova, Jacqueline Scholl, Laurie Josephs, Andrew J Quinn, Mark W Woolrich, Charlotte J Stagg

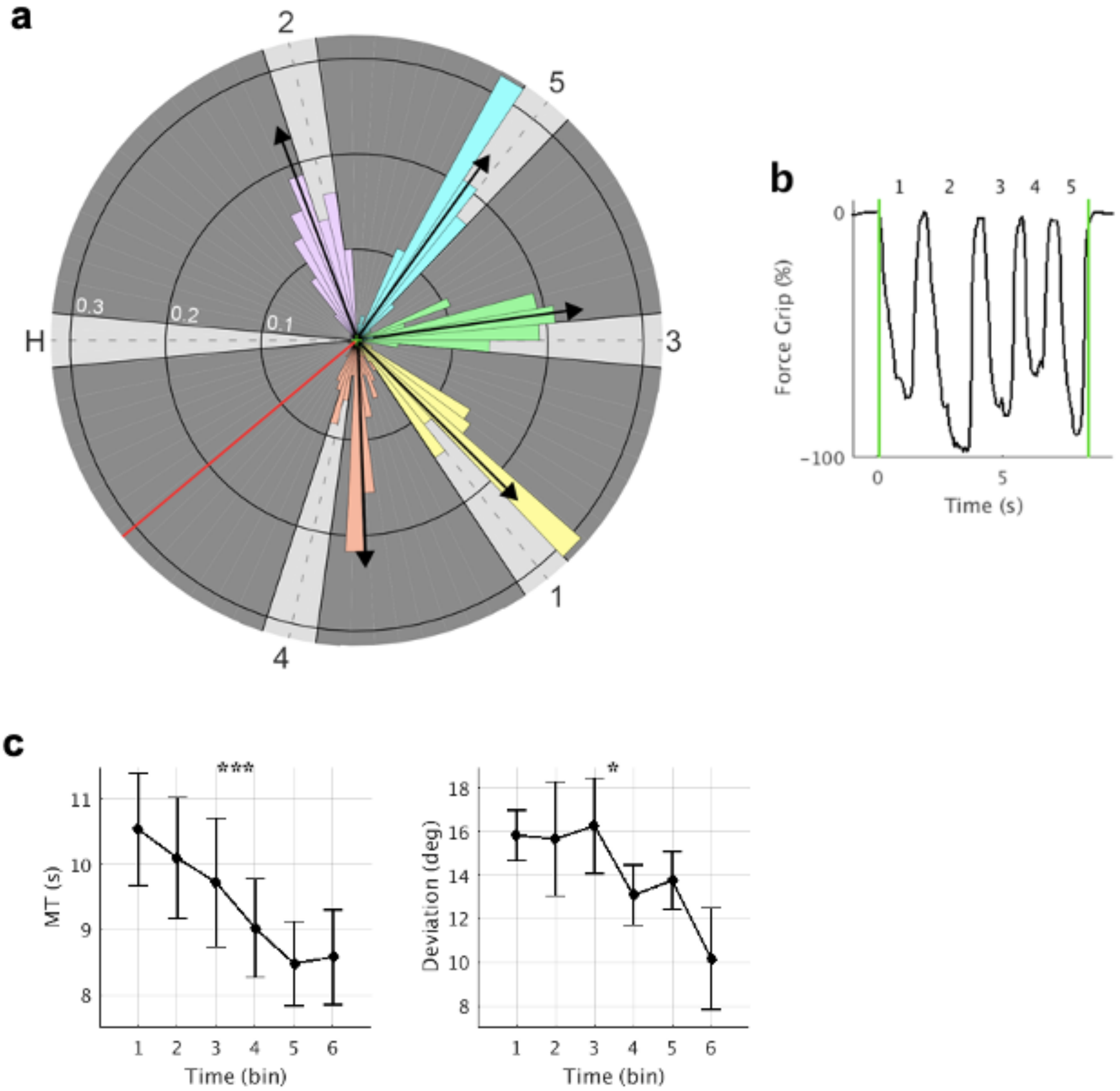

SI Fig. 1 *Motor learning task (ML task 2) of Experiment 2*
a) Task design overlaid with the distribution of accuracy. Participants saw the dark grey circle with its light grey numbered gates and a red cursor over the whole time of the visuo-motor task. Rotation of the on-screen cursor (red line) was controlled by squeezing (anti-clockwise) and relaxing (clockwise) a force transducer with the right hand. The goal of the task was to move the cursor quickly and accurately between the start/end position (Home) and a numbered order of gates (Home-1-Home-2-Home-3-Home-4-Home-5-Home). Here, the visual display shown to the participants is overlaid with the accuracy histograms of the circular distance between the reversal point of the cursor and the centre of the approached gate. The direction of the arrow indicates the average accuracy for each gate. Note that the distance to the centre of the circle and the centre of the gates are not displayed to the participants.
b) Single-trial force data epoched from 1 s before movement onset to 1 s after movement offset. Green lines indicate movement onset and offset.
c) Participants improved in terms of decreased movement time (MT, left) and increased accuracy (less deviation, right) with practice. Error bars represent standard error across individuals.

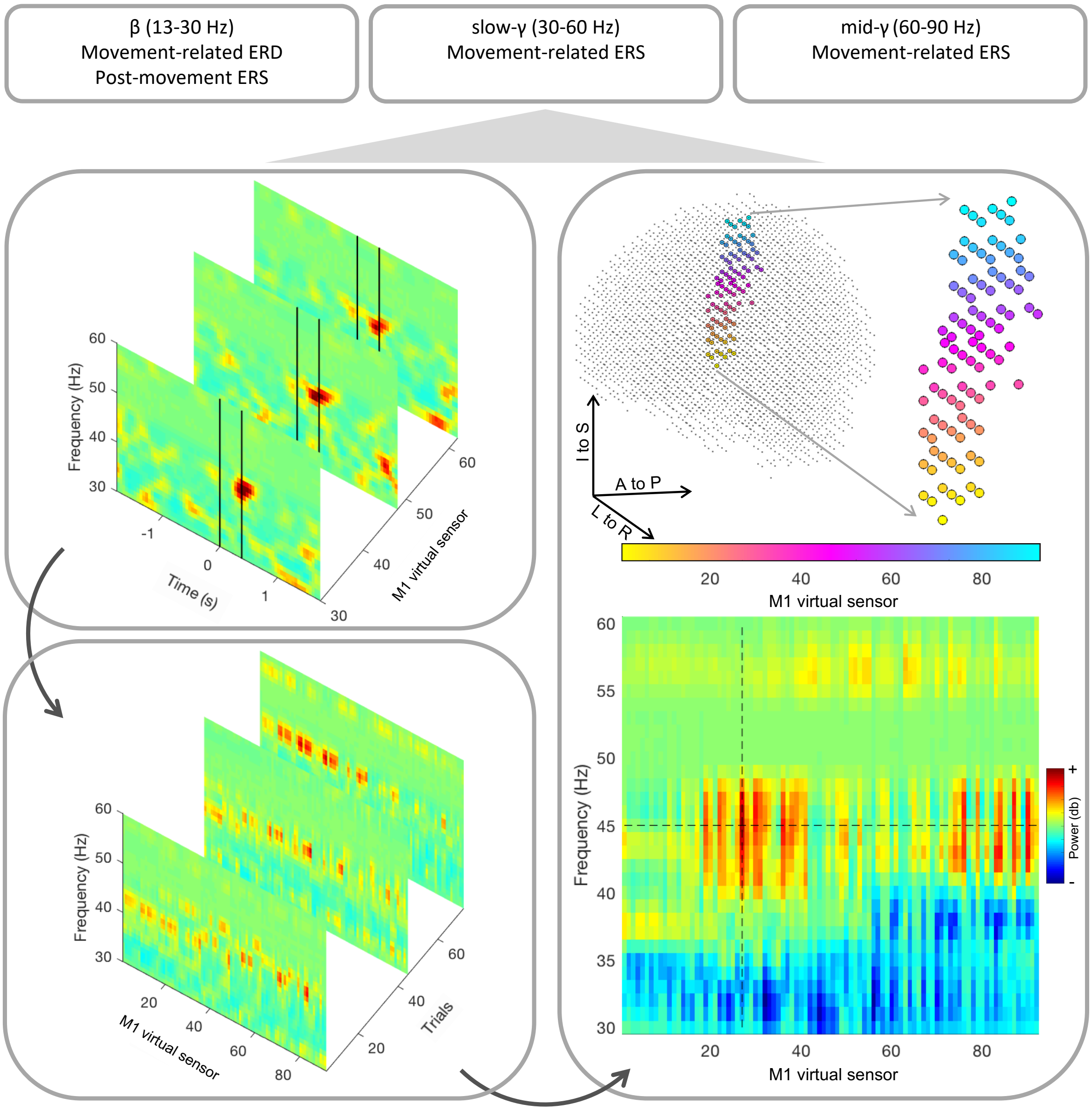

SI Fig. 2 *Pipeline used to identify the individuals' peak frequency. The procedure was identical for β ERD, β ERS, slow-γ ERS, mid-γ ERS. Details are exemplary illustrated for slow-γ ERS.*

(top left panel) Power for one individual trial in 3D (time [x axis], frequency [y axis], space [z axis]). For each trial the power from movement onset to movement offset (movement offset to movement offset + 1 s for β ERS), as determined by EMG and indicated by the black vertical lines, was averaged over time. Averaging over time reduces data from 4D (time, frequency, space, trial) to 3D (space, frequency, trial).

(bottom left panel) Power in 3D (space [x axis], frequency [y axis], trial [z axis]) averaged over time. Averaging over trials reduces data from 3D (space, frequency, trial) to 2D (space, frequency).

(right panel) Power in 2D (space [x axis], frequency [y axis]) averaged over time and trials. The maximum (minimum for β ERD) of these 2D power data indicates the individuals' power, peak frequency (dashed horizontal line) and virtual sensor within the sensorimotor cortex (dashed vertical line). Virtual sensors are ordered based on MNI z-coordinates and color-coded accordingly (e.g., small index = yellow = inferior).

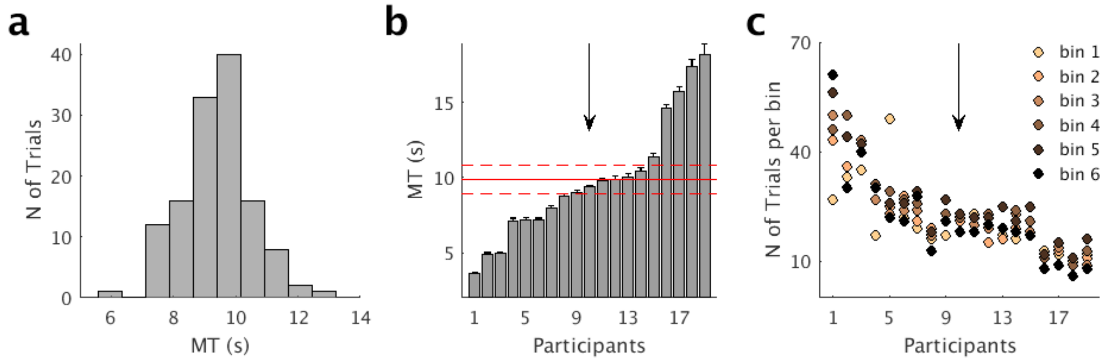

SI Fig. 3 *Intra- and inter-individual differences in movement time and number of trials for the motor learning task (ML task 2) of Experiment 2*

a) Distribution of movement time (MT) for one representative individual.

b) Mean movement time per individual with error bars reflecting standard error. Individuals are sorted based on mean movement time. Horizontal red lines indicate group average plus/ minus one standard error. Arrow indicates the individual displayed in (a).

c) Number of trials per bin for each participant separately (Supplementary Methods for details). Individuals are sorted based on mean movement time. Arrow indicates the individual displayed in (a).

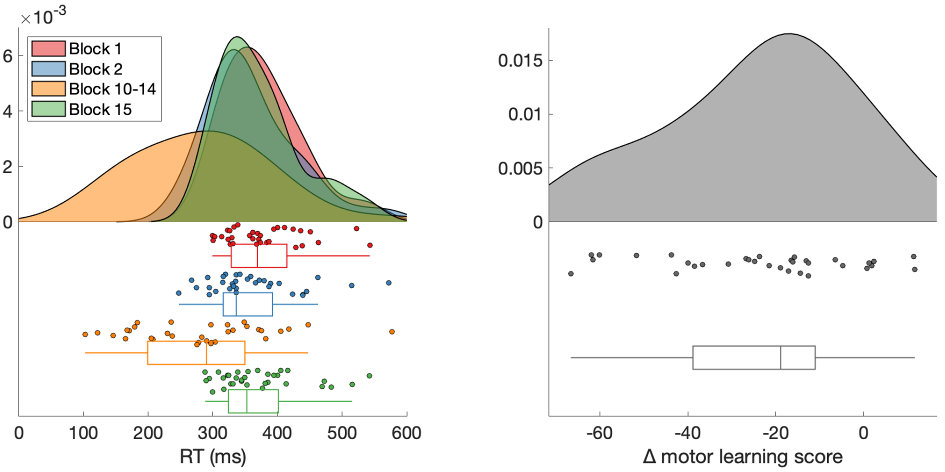

SI Fig. 4 *Reaction time (RT) and change in motor learning score for the motor learning task (ML task 1) in Experiment 1.*

(left) Distribution of the mean reaction time per subject separately for block 1 (random sequence block), block 2 (first sequence block), the average of block 10 – 14 (sequence blocks), i.e., when the learning plateaued (Stagg et al., 2011), and block 15 (random sequence block). Displayed are the distribution across subjects (top), individual subject data, and data as box blot (bottom).

(right) Distribution of the motor learning score, i.e., percentage change from the RT in the first sequence block (block 2) to blocks 10-14 when the learning plateaued (Stagg et al., 2011), across subjects (top), individual subject data, and data as box blot (bottom).
